# Blood Biomarkers of Chronic Inflammation in Gulf War Illness

**DOI:** 10.1371/journal.pone.0157855

**Published:** 2016-06-28

**Authors:** Gerhard J. Johnson, Billie C. S. Slater, Linda A. Leis, Thomas S. Rector, Ronald R. Bach

**Affiliations:** 1 Department of Veterans Affairs Health Care System, Minneapolis, Minnesota, 55417, United States of America; 2 Department of Medicine, University of Minnesota, Minneapolis, Minnesota, 55455, United States of America; Indiana School of Medicine, UNITED STATES

## Abstract

**Background:**

More than twenty years following the end of the 1990–1991 Gulf War it is estimated that approximately 300,000 veterans of this conflict suffer from an unexplained chronic, multi-system disorder known as Gulf War Illness (GWI). The etiology of GWI may be exposure to chemical toxins, but it remains only partially defined, and its case definition is based only on symptoms. Objective criteria for the diagnosis of GWI are urgently needed for diagnosis and therapeutic research.

**Objective:**

This study was designed to determine if blood biomarkers could provide objective criteria to assist diagnosis of GWI.

**Design:**

A surveillance study of 85 Gulf War Veteran volunteers identified from the Department of Veterans Affairs Minnesota Gulf War registry was performed. All subjects were deployed to the Gulf War. Fifty seven subjects had GWI defined by CDC criteria, and 28 did not have symptomatic criteria for a diagnosis of GWI. Statistical analyses were performed on peripheral blood counts and assays of 61 plasma proteins using the Mann-Whitney rank sum test to compare biomarker distributions and stepwise logistic regression to formulate a diagnostic model.

**Results:**

Lymphocyte, monocyte, neutrophil, and platelet counts were higher in GWI subjects. Six serum proteins associated with inflammation were significantly different in GWI subjects. A diagnostic model of three biomarkers—lymphocytes, monocytes, and C reactive protein—had a predicted probability of 90% (CI 76–90%) for diagnosing GWI when the probability of having GWI was above 70%.

**Significance:**

The results of the current study indicate that inflammation is a component of the pathobiology of GWI. Analysis of the data resulted in a model utilizing three readily measurable biomarkers that appears to significantly augment the symptom-based case definition of GWI. These new observations are highly relevant to the diagnosis of GWI, and to therapeutic trials.

## Introduction

From August 2, 1990 to July 31, 1991 approximately 697,000 United States military personnel were deployed to the Kuwaiti Theater of Operations during Operation Desert Shield and Operation Desert Storm (Gulf War)[1]. Many veterans of this conflict—estimated to be approximately 300,000 based on data from a recent survey of Gulf War veterans [2]—now suffer from an unexplained chronic multi-symptom disorder known as Gulf War Illness (GWI). The symptoms most frequently associated with GWI are widespread pain, unexplained fatigue, and cognitive difficulties. Comprehensive reviews of GWI have been published by the Research Advisory Committee on Gulf War Veterans’ Illnesses [3, 4] and the Institute of Medicine [1, 5, 6].

The Department of Veterans Affairs (VA) Office of Public Health (OPH) has conducted survey studies of the mental and physical health of a population-based cohort of 30,000 Gulf War and Gulf War era Veterans [2, 7–9]. The most significant health-related difference revealed by these studies was the higher prevalence of unexplained chronic multi-symptom illnesses in the deployed veterans group. Ten years post-deployment the difference was 28.9% vs 15.8% (adjusted odds ratio = 2.16) [8]. Fourteen years post-deployment the difference was 36.5% vs 11.7% (adjusted risk ratio = 3.05) [9]. Twenty years post-deployment the difference was 43.9% vs 20.3% (adjusted odds ratio = 3.06) [2]. Thus a chronic unexplained multi-symptom illness is the signature health-related outcome of the 1990–1991 Gulf War and the incidence of GWI continues to increase.

Multiple studies of Gulf War and Gulf War era Veterans from several countries consistently found higher rates and greater severity of symptoms in deployed than in non-deployed veterans. However, the variability of symptoms among the deployed, the presence of the same symptoms in non-deployed veterans, and multiple biases, prompted skepticism by authors of an Institute of Medicine review [1] regarding the representativeness of these studies. This review concluded that the data available did not support definition of a unique syndrome. They further cautioned that it was unlikely that factor analysis or cluster analysis of symptoms could produce a satisfactory case definition of GWI [1]. Therefore, we searched for objective parameters to augment current symptomatic diagnostic criteria.

Evidence of immunological aberrations in veterans with GWI has been published by other research groups [10–19]. However, no consensus has been reached regarding immunological criteria for the diagnosis of GWI [17]. Kelsall, et al [20] reported evidence of inflammation (elevated erythrocyte sedimentation rate, C-reactive protein, or leucocyte count) in Australian Veterans of the Gulf War who had multisymptom illness, but only pooled biomarker data was published. A previous study carried out in our laboratory also found higher blood CRP, plus elevated platelet counts and thromboxane analog-stimulated platelet aggregation, in deployed veterans with GWI compared to deployed veterans without GWI [21]. The results of this study stimulated evaluation of a larger cohort of Gulf War Veterans to determine if significant blood biomarker differences exist between veterans with GWI and without GWI as defined by accepted symptomatic criteria [22].

## Methods

### IRB Approval

The study was approved by the Human Studies Subcommittee of the Research Committee of the Minneapolis Veterans Affairs Health Care System (MVAHCS), and by the U.S. Army Medical Research and Materiel Command IRB.

### Subjects

Five hundred deployed veterans of the Gulf War enrolled in the Department of Veterans Affairs Minnesota Gulf War Registry were invited to participate in the study by means of an IRB–approved letter. Eighty six veterans replied, and 85 entered the study between 2010 and 2013. Veterans who volunteered for the study were interviewed in person. Written informed consent was obtained. A structured interview assessed their health status, and blood samples were obtained.

### Inclusion and Exclusion Criteria

Deployed Gulf War Veterans were eligible for entry into the study unless they had cancer, liver disease, acute or chronic inflammatory states, or other major chronic illness including chronic fatigue syndrome and fibromyalgia. Post-traumatic stress disorder (PTSD) did not exclude subjects. All 85 of the Gulf War Veterans who volunteered for the study were eligible; so none were excluded.

### Subject Classification

All subjects completed a symptom questionnaire that contained the elements of the case definition of the chronic multisystem illness characteristic of GWI developed by Fukuda and colleagues [22], also referred to as the CDC-10 survey. This survey included three categories with nine subcategories: 1) fatigue; 2) mood-cognition (depression, anxiety, moodiness, memory problems, difficulty with words, difficulty sleeping); 3) musculoskeletal (muscle pain, joint pain, joint stiffness). Subjects were considered to have GWI, and were classified as GWI+ if, a) they had one or more chronic symptoms from at least 2 of 3 of the case-defining symptom categories—fatigue, mood-cognition, and musculoskeletal pain; b) the symptoms began during or after the 1990–1991 Gulf War; and c) symptoms were present for at least 6 months. Subjects without case-defining symptoms or symptoms in only one category were considered not to have GWI, and were classified as GWI-.

### Blood Sample Collection and Preparation

Non-fasting, peripheral venous blood was collected into 4.0 ml Vacutainer^®^ vacuum tubes (BD, Franklin Lakes, NJ, USA) containing 7.2 mg K2 EDTA for evaluation of blood cell and plasma protein components. One tube was sent to the MVAHCS Clinical Laboratory for a complete blood count with differential leucocyte count. A second tube was used to prepare platelet-free plasma (PFP). Platelet-poor plasma (PPP) was isolated from whole blood by centrifugation at 3,200 rpm for 15 min (1,770g) at room temperature (RT). The PPP layer was carefully removed, and the PPP was centrifuged at 3,200 rpm for 15 min at RT repeatedly if necessary until the platelet count was ≤1/μl (Beckman Coulter AcTdiff 2 counter). PFP aliquots were snap frozen on dry ice, and stored at -80°C.

### Plasma Proteomic Analysis

Frozen PFP samples were sent to Myriad RBM, Austin, TX, for proteomic analysis performed by quantitative multiplexed immunoassays using a Multi-Analyte Profile (MAP) platform [23, 24]. HumanMAP^®^ v.1.6 analysis was programmed to measure 88 plasma proteins (Table 1). Twenty-seven of the antigens were not included in the statistical analyses because measurable protein values were not detected in at least 90% of the subjects (Table 1).

**Table 1:** HumanMAP® v.1.6 analysis by Myriad RBM, Austin TX, USA.

ANTIGEN	N*	ANTIGEN	N
Alpha-2-macroglobulin	85	Interleukin-4	36
Alpha-1-Antitrypsin	85	Interleukin-5	67
Adiponectin	85	Interleukin-6	55
Alpha-Fetoprotein	85	Interleukin-7	56
Apolipoprotein(a)	84	Interleukin-8	84
Apolipoprotein A-I	85	Interleukin-10	79
Apolipoprotein C-III	85	Interleukin-12p40	48
Apolipoprotein H	85	Interleukin-12p70	14
Beta-2-Microglobulin	85	Interleukin-13	71
Brain-Derived Neurotrophic Factor	85	Interleukin-15	60
Complement C3	85	Interleukin-16	85
Cancer Antigen 125	66	Interleukin-18	85
Cancer Antigen 19–9	81	Insulin	84
Calcitonin	82	Leptin	85
CD 40 antigen	85	Lymphotactin	2
CD40 Ligand	85	Monocyte Chemotactic Protein 1	85
Carcinoembryonic Antigen	85	Macrophage-Derived Chemokine	85
Creatine Kinase-MB	84	MIP-1 alpha	85
C-Reactive Protein	84	MIP-1 beta	85
Epidermal Growth Factor	41	Matrix Metalloproteinase-2	85
ENA-78	85	Matrix Metalloproteinase-3	27
EN-RAGE	60	Matrix Metalloproteinase-9	85
Eotaxin-1	73	Myeloperoxidase	78
Erythropoietin	34	Myoglobin	85
Endothelin-1	43	Plasminogen Activator Inhibitor 1	85
Fatty Acid-Binding Protein 3	79	Prostatic Acid Phosphatase	85
Factor VII	85	PAPPA	72
Fibroblast Growth Factor Basic	16	Prostate-Specific Antigen, Free	85
Fibrinogen	85	RANTES	85
Ferritin	85	Serum Amyloid P-Component	85
GCSF	73	Stem Cell Factor	85
Growth Hormone	69	SGOT	84
GM-CSF	23	Sex Hormone-Binding Globulin	85
Haptoglobin	84	Thyroxine-Binding Globulin	85
Intercellular Adhesion Molecule 1	85	Tissue Factor	29
Interferon gamma	29	Tissue Inhibitor of Metalloproteinases 1	85
Immunoglobulin A	84	Tumor Necrosis Factor alpha	77
Immunoglobulin E	83	Tumor Necrosis Factor beta	27
Immunoglobulin M	85	Tumor Necrosis Factor Receptor 2	85
Interleukin-1 alpha	52	Thrombopoietin	80
Interleukin-1 beta	24	Thyroid-Stimulating Hormone	84
Interleukin-1 receptor antagonist	77	Vascular Cell Adhesion Molecule-1	85
Interleukin-2	6	VEGF	85
Interleukin-3	80	vonWillebrand Factor	85

* number of subjects which yielded protein concentrations above the lower limit of detection

### Statistical Analyses

The Mann-Whitney rank sum test was used to compare the distribution of each biomarker between the GWI+ and GWI- groups. The c-statistic is reported as a measure of discrimination.

Stepwise logistic regression was employed first to identify cell counts that may provide independent discriminatory information. Other biomarkers were then tested one by one to see if they provided additional discriminatory information. Given the modest sample, a p-value <0.10 was considered significant without correction for the number of comparisons in this effort to identify diagnostic biomarkers.

## Results

### Demographic Data

Characteristics of the 57 (67%) GWI+ and 28 (33%) GWI- veterans enrolled in this study are presented in Table 2. The subjects were predominantly male (95%), white (93%), and middle aged (median 46–48 years). The median weights (p = 0.249) and BMIs (p = 0.236) of the GWI+ and GWI- groups were not significantly different. PTSD had been diagnosed in 17.5% of GWI+ subjects (all male), but in none of the GWI- subjects. Fourteen percent of the GWI+ subjects and 4% of GWI- subjects were being treated with antidepressants, or other psychotropic medications at the time of study.

**Table 2:** Characteristics of Subjects at Time of Study.

Characteristics	GWI+	GWI-
**Number of Participants (n)**	57	28
**Age**		
**Age, years (median)**	46	48
**Age, years (range)**	(38–68)	(38–70)
**BMI**		
**BMI (median)**	31	28
**BMI (range)**	(19–46)	(22–47)
**BMI <30 (median)**	27	26
**BMI ≥30 (median)**	34	36
**Weight**		
**Weight, lbs. (median)**	220	200
**Weight, lbs. (range)**	(125–360)	(130–320)
**Gender**		
**Female**	3	1
**Male**	54	27
**Ethnic Origin**		
**Black**	2	2
**White**	53	26
**Hispanic**	2	0
**Symptoms**		
**None**	0	13
**Single**	0	15
**Multiple**	57	0
**Cognition**	57	6
**Fatigue**	52	6
**Pain**	48	1
**Use of Nicotine**	42%	21%
**Concomitant Medications**		
**NSAIDS**	58%	57%
**OTC supplements**	32%	61%
**Statins**	18%	32%
**Antidepressants/other psych meds**	14%	4%
**Opiates**	7%	4%

### Symptomatology

By definition, 100% of GWI+ subjects had multiple symptoms compared to none of the GWI- subjects (Table 2). Forty six percent of GWI- subjects had no relevant case- defining symptoms, and 53% had a single symptom. Among GWI+ subjects cognitive disturbances were present in 100%, fatigue in 91%, and pain in 84%. Twenty one per cent of GWI- subjects had cognitive dysfunction, 21% had fatigue, and 4% had pain.

### Hematological Data

Compared to GWI- subjects, the distributions of peripheral blood lymphocyte, monocyte, neutrophil, and platelet counts were higher in GWI+ subjects (Table 3).

**Table 3:** Blood Cell Counts for GWI+ and GWI- Veterans.

Blood Cell Type	GWI+ (n = 57), Cells x10^3^/μl	GWI- (n = 28), Cells x 10^3^/μl	c-statistic*	p-value^†^
**White Blood Cells**	6.54 (5.55–7.74)^‡^	5.66 (4.33–6.56)	0.69	0.005
**Lymphocytes**	1.82 (1.49–2.39)	1.50 (1.35–1.76)	0.70	0.004
**Monocytes**	0.51 (0.42–0.66)	0.40 (0.35–0.52)	0.68	0.009
**Neutrophils**	3.91 (3.27–4.87)	3.29 (2.58–3.96)	0.67	0.010
**Platelets**	222 (201–259)	205 (164–240)	0.65	0.030

* A value of 0.50 would indicate no discrimination, 1.0 would be perfect discrimination of those with GWI.

† Mann-Whitney test

‡ Median (interquartiles)

The lymphocyte counts were slightly better at discriminating the GWI groups. The distributions of neutrophil to lymphocyte ratios were not significantly different.

### Proteomic Analysis

The most discriminating plasma proteomic variables are summarized in Table 4.

**Table 4:** Proteomic Analysis of Plasmas from GWI+ and GWI- Veterans.

Plasma Proteins	GWI+ (n = 57)	GWI- (n = 28)	c-statistic*	p-value^†^
**C-Reactive Protein (CRP), μg/ml**	2.1^‡^	1.2	0.65	0.03
**C-Reactive Protein (CRP), μg/ml (range)**	(1.0–4.2)	(0.7–2.8)		
**Leptin, ng/ml**	9	5.4	0.63	0.05
**Leptin, ng/ml (range)**	(4.5–13.8)	(2.8–11.1)		
**Matrix Metalloproteinase-9 (MMP-9), ng/ml**	103	90	0.64	0.03
**Matrix Metalloproteinase-9 (MMP-9), ng/ml (range)**	(79–141)	(66–116)		
**Brain-Derived Neurotrophic Factor (BDNF), ng/ml**	1.3	0.81	0.66	0.02
**Brain-Derived Neurotrophic Factor (BDNF), ng/ml (range)**	(0.83–1.86)	(0.36–1.38)		
**Matrix Metalloproteinase-2 (MMP-2), ng/ml**	2130	2430	0.69	0.004
**Matrix Metalloproteinase-2 (MMP-2), ng/ml (range)**	(1770–2590)	(2250–3175)		
**Heart-Fatty Acid-Binding Protein (H-FABP), ng/ml**	1.8	2.8	0.66	0.02
**Heart-Fatty Acid-Binding Protein (H-FABP), ng/ml (range)**	(0.9–3.1)	(1.9–3.7)		

* A value of 0.50 would indicate no discrimination, 1.0 would be perfect discrimination of those with GWI.

† Mann-Whitney test

‡ Median (interquartiles)

Six statistically significant differences (p<0.05) between GWI+ and GWI- subjects were identified. Plasma C-reactive protein (CRP), leptin, brain-derived neurotrophic factor (BDNF), and matrix metalloproteinase-9 (MMP-9) were significantly higher in the GWI+ group. Heart-type fatty acid binding protein (H-FABP) and matrix metalloproteinase-2 (MMP-2) were significantly lower in the blood of GWI+ subjects. The level of detection of cytokines, such as IL-1α, IL-1β, IL-2, IL-6 and interferon γ, was well below the criteria for analysis (<10% of subjects with undetectable levels).

### Diagnostic Model

**Fig 1:**
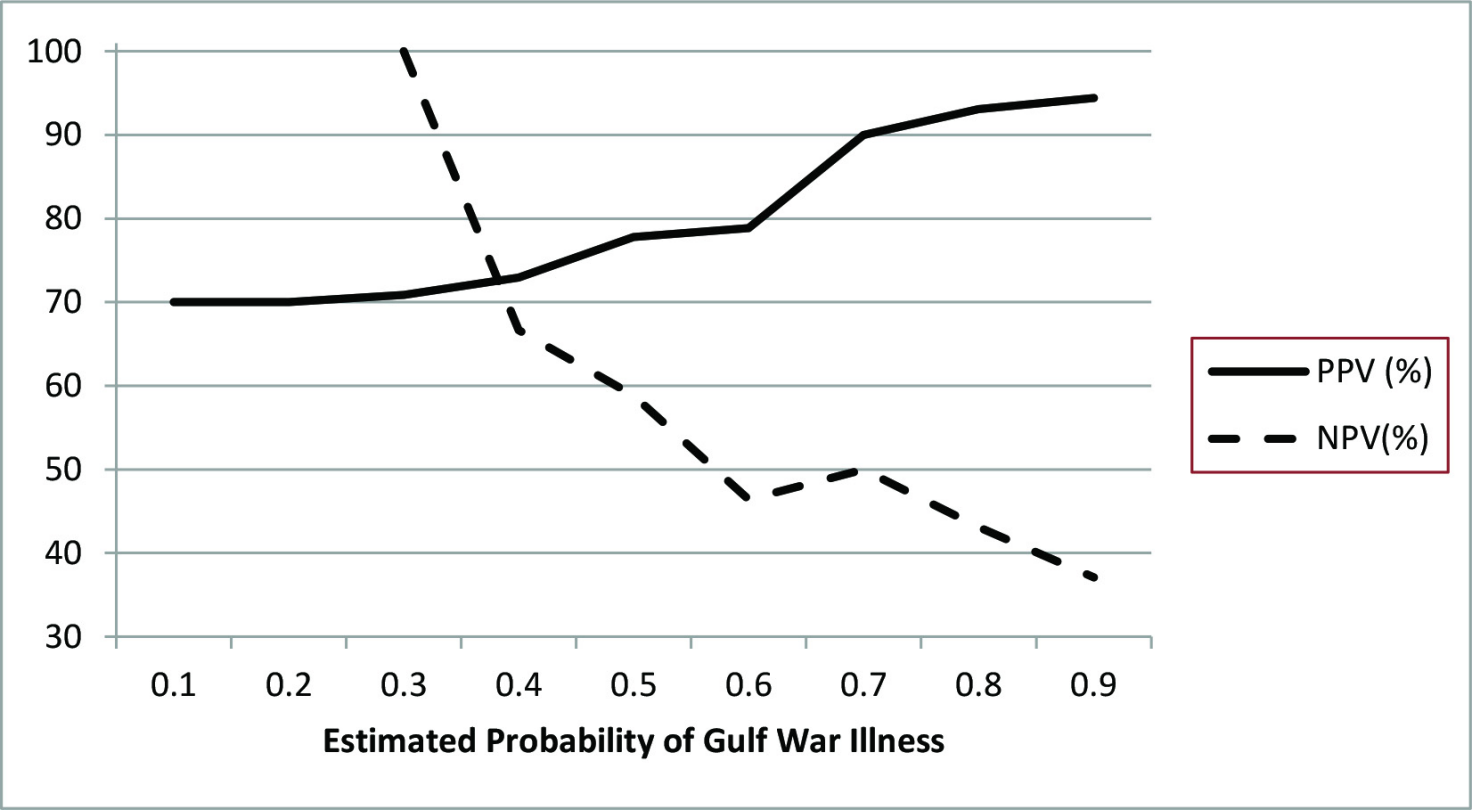
The positive predictive value (PPV) and negative predictive value (NPV) of a multivariable model of GWI.

Stepwise multivariable logistic regression led to a diagnostic model comprised of three biomarkers—lymphocytes, monocytes, and CRP. We estimated the probability of having GWI using this fitted model. A positive diagnosis of GWI was defined as a model-estimated probability exceeding 0.70 (70%). For subjects with a model-predicted probability of >70% [a criterion met by 40/80 (50%) of the sample] the positive predictive value of the confirmatory diagnostic model was 90% (95% confidence interval 76–97%) for diagnosing GWI. The corresponding negative predictive value for ruling out GWI was 50% (95% confidence interval 34–66%; Fig 1). The model c-statistic was 0.77 (95% confidence interval 0.67–0.88; p = 0.05).

Since obesity occurred in GWI+ and GWI- groups, a characteristic of Gulf War era veterans described previously [2, 20, 25], the potential influence of obesity on the diagnostic model was analysed. BMI was weakly correlated with lymphocytes (r = 0.22) and monocytes (r = 0.13), and moderately correlated with CRP (r = 0.43); however, BMI did not reach the specified p value of 0.1 to enter the model after the addition of lymphocytes and monocytes. Therefore, BMI did not add to the discrimination of the model. Similarly, leptin was highly correlated with BMI (r = 0.78), but the addition of leptin did not add discriminating information to the lymphocyte, monocyte model.

## Discussion

Long after the end of the 1990–1991 Gulf War many veterans of this conflict are ill with an unexplained chronic, multi-system disorder recognized by the Department of Veterans Affairs as GWI [2, 26]. This disorder is characterized by an incomplete understanding of its etiology and pathophysiology, and a case definition based only on symptoms [22, 27]. The absence of objective diagnostic criteria is a substantial barrier to clinical diagnosis and research. Despite research by multiple investigators, readily measurable parameters that would permit an objective diagnosis of GWI have not previously been identified. The results of the current study provide evidence of alterations in a number of blood parameters that are readily measurable in routine clinical laboratories. We found white blood cell counts and blood biomarkers related to inflammation could discriminate groups that did or did not meet the current symptom-based criteria for case definition of GWI. Our observations are consistent with conclusions expressed in recent literature reviews that immune dysregulation/neuroinflammation are components of the pathobiology of GWI [28, 29]. Appropriate assays for the presence of chronic inflammation could provide objective evidence that would facilitate the diagnosis of GWI+.

Alterations of leucocyte counts, particularly the neutrophil to lymphocyte ratio, have recently been reported to have prognostic significance in a wide range of diseases [30–33]. An elevated neutrophil to lymphocyte ratio, often found to correlate with CRP or IL6 levels [30, 34, 35] has been interpreted as evidence of an inflammatory component of the disorder studied. In contrast to other inflammatory conditions, we did not observe an increase in the neutrophil to lymphocyte ratio because the lymphocyte count was elevated rather than decreased as described in other studies.

Support for an inflammatory component of GWI is provided by the significantly higher levels of plasma CRP detected in GWI+ veterans. CRP is an acute phase plasma protein synthesized in the liver which rises rapidly in response to infection or tissue injury [36]. CRP is frequently employed as a biomarker of IL-6-mediated inflammation, and it may also augment inflammation. CRP exists in two distinct protein confirmations. Native pentameric CRP is the circulating precursor of monomeric CRP which is strongly proinflammatory [37].

In some inflammatory disorders, CRP is highly elevated, but in other disorders modest elevations of CRP have been found to be indicators of chronic inflammation with prognostic significance [38–41]. In coronary artery disease CRP concentrations found in the general population (1–3 μg/ml) predict increased cardiovascular mortality [41].

Leptin, an adipokine produced primarily by white fat tissue, is another biomarker linked to inflammation, and found in the current study to be elevated in GWI+ veterans. Leptin production is elevated in experimental inflammation and in human autoimmune diseases [42, 43]. Leptin is known to cross the blood-brain barrier and to interact with cells in the hypothalamus, arcuate nucleus, and endothelium, and with leucocytes. These interactions have been shown to result in prolonged neuroinflammation with behavioral changes in experimental animals [43]. A leptin antagonist mutant demonstrated benefit in experimental autoimmune inflammatory bowel disease [44]. Leptin also affects hematopoiesis [45–49]. It may have contributed to the elevated lymphocyte counts observed in GWI+ subjects, and thus explain why leptin was not an independent correlate of GWI+. Leptin has also been demonstrated to interact with CRP. In vitro studies found leptin to stimulate the expression of CRP by human hepatocytes. In addition, CRP bound to leptin and directly inhibited its binding to its receptors [50].

BDNF, another plasma protein elevated in GWI+ subjects, may also be a biomarker of inflammation in GWI. BDNF is a neurotrophin which functions as a major regulator of synaptic plasticity and neurogenesis in the central nervous system [51]. Blood BDNF derives primarily from the brain [52], and has been observed in several diseases accompanied by inflammation—rapid cycling bipolar disorder [53], Alzheimer’s disease [54], and fibromyalgia [55]. Multiple animal studies indicate that overexpression of BDNF following nerve injury or peripheral inflammation stimulates synaptic changes that contribute to chronic pain [56]. Thus, inflammation-induced BDNF could be a mediator of cognitive impairment and chronic pain in GWI. Neither serum nor plasma BDNF correlate with body weight [57] or obesity [58].

Matrix metalloproteinases (MMPs) are endopeptidases that participate in tissue extracellular matrix degradation and remodeling. MMP activation has been observed in inflammatory and neurodegenerative disorders [59, 60]. Elevation of plasma MMP-2, MMP-9 or both have been observed in coronary heart disease [61], polypoidal choroidal vasculopathy [62] and Japanese encephalitis [63]. We observed higher median MMP-9, but lower median MMP-2 in GWI+ subjects than in GWI- subjects. The reason for the opposing direction of change is unexplained, but precedent exists for differential patterns of MMP-2 and MMP-9 gene expression in coronary heart disease [61], chronic obstructive pulmonary disease [64], and thoracic aortic aneurysm [65].

H-FABP is a fatty acid binding protein expressed primarily in the heart. Release of H-FABP into the blood occurs during cardiac ischemia, strenuous exercise, and neurodegenerative disorders, but low levels have been reported to occur in patients with Down syndrome [66]. Decreased H-FABP has been postulated to protect against atherosclerosis. In the current study median plasma H-FABP was significantly lower in GWI+ subjects than in GWI- subjects. Possibly relevant to the lower blood levels in GWI+ subjects is the observation that fatty acid binding protein mRNA was substantially decreased in hamster skeletal muscle and heart muscle by LPS-induced inflammation [67]. It is possible that blood H-FABP is suppressed by inflammation in GWI, and that the suppression is modulated by elevated blood leptin [68].

Although inflammatory mediators are implicated, the precise stimuli for the elevations of blood cell counts, CRP, leptin and the alterations in other blood proteins could not be determined by the current study, and the relationship of these parameters to the symptoms experienced by the subjects with GWI can only be hypothesized. However, observations of others noted above suggest that some of the patient’s symptoms may be caused by or accentuated by CRP, leptin, BDNF or other inflammation-related blood proteins.

This study has strengths and limitations that must be considered in assessing its significance. A strength of the study is the classification of subjects deployed to the Gulf War based on accepted case-definition criteria. Another strength of the study is the evaluation of blood parameters that are readily available in clinical laboratories. The limitations of the study include small sample size, restricted geographic, ethnic, and sex composition of the study subjects, assay of blood parameters only once, some plasma protein assays, including cytokines, considered inevaluable due to a high percentage of assays below the level of detection, overlap of biomarker distributions within the normal range, absence of correction for multiple comparisons, a limited number of blood proteins found to be positively related to GWI+ status, and the absence of a confirmation cohort study.

Despite these limitations, the diagnostic model yielded a high positive predictive value for the 50% of the study participants who had an estimated probability of GWI of 70%, although it is recognized that this estimate may be optimistic because the diagnostic model was fit to these particular data. Although the positive association of 6/61 plasma proteins with GWI+ subjects may have occurred by chance, the close functional and biochemical relationships of these proteins suggest that the differences were not random events. Also, the observation that the subsequent addition of other inflammation-related proteins did not improve the predictive probability of the model above that provided by CRP is consistent with a functional relationship of these parameters.

Although not a deficiency of the study, the issue of obesity is a confounding variable for interpretation of the results of the current study as well as other studies of GWI. The median body weights and BMIs of GWI+ and GWI- subjects were not significantly different, and BMI did not enter into the multivariable diagnostic model. Both groups included obese subjects. Similar obesity observations were found in studies of American [2, 25] and Australian Gulf War Veterans [20]. These studies found no differences in BMI of subjects with GWI and those in comparison groups of veterans who were either non-deployed or deployed to areas other than the Persian Gulf. Coughlin, et al [25] found no associations between BMI and unexplained multisystem illness in multivariate analysis. Kelsall, et al [20] found a relationship between laboratory parameters of inflammation and multisystem illness, but BMI was not related to multisystem illness [20]. Dursa, et al [2] reported average BMIs of 29.8 for Gulf War Veterans and 29.7 for Gulf War era Veterans 20 years after the Gulf War. These figures are quite similar to those we observed in GWI+ subjects (BMI 31) and GWI-subjects (BMI 28). Therefore, elevation of some of the inflammatory parameters observed in the current study may be attributable to obesity, but obesity does not explain the differences between the GWI+ and GWI- groups, and does not diminish the predictive value of the diagnostic model.

In summary, the results of the current study support the hypothesis that chronic inflammation is a component of the pathophysiology of GWI. Multivariable logistic regression analysis resulted in a model with a high positive predictive value for GWI in subjects with symptoms considered to be significant by current case definition criteria. This diagnostic model requires validation in other samples of Gulf War Veterans. A clinical trial that will further evaluate inflammatory parameters and the efficacy of anti-inflammatory therapy in GWI is in progress at our institution (Gulf War Illness Inflammation Reduction Trial, ClinicalTrials.gov #NCT02506192). The results of this clinical trial will provide valuable data to further evaluate the utility of measuring inflammatory biomarkers in the diagnosis of GWI, but validation by studies of other cohorts of veterans with GWI is required.
